# Attention-Guided Probabilistic Diffusion Model for Generating Cell-Type-Specific Gene Regulatory Networks from Gene Expression Profiles

**DOI:** 10.3390/genes16111255

**Published:** 2025-10-24

**Authors:** Shiyu Xu, Na Yu, Daoliang Zhang, Chuanyuan Wang

**Affiliations:** 1 Department of Bioinformatics, School of Life Sciences, Xuzhou Medical University, Xuzhou 221004, China; xushiyu163@163.com; 2 School of Life Sciences, Westlake University, Hangzhou 310030, China; yuna@westlake.edu.cn; 3 Institute of Science and Technology for Brain-Inspired Intelligence, MOE Key Laboratory of Computational Neuroscience and Brain-Inspired Intelligence, MOE Frontiers Center for Brain Science, Fudan University, Shanghai 200032, China; zhangdaoliang@fudan.edu.cn; 4 Department of Biomedical Engineering, School of Control Science and Engineering, Shandong University, Jinan 250061, China

**Keywords:** gene regulatory network, diffusion generative model, hybrid-attention mechanism, gene expression profile, mouse aging progression

## Abstract

Gene regulatory networks (GRN) govern cellular identity and function through precise control of gene transcription. Single-cell technologies have provided powerful means to dissect regulatory mechanisms within specific cellular states. However, existing computational approaches for modeling single-cell RNA sequencing (scRNA-seq) data often infer local regulatory interactions independently, which limits their ability to resolve regulatory mechanisms from a global perspective. Here, we propose a deep learning framework (Planet) based on diffusion models for constructing cell-specific GRN, thereby providing a systems-level view of how protein regulators orchestrate transcriptional programs. Planet jointly optimizes local network structures in conjunction with gene expression profiles, thereby enhancing the structural consistency of the resulting networks at the global level. Specifically, Planet decomposes GRN generation into a series of Markovian evolution steps and introduces a Triple Hybrid-Attention Transformer to capture long-range regulatory dependencies across diffusion time-steps. Benchmarks on multiple scRNA-seq datasets demonstrate that Planet achieves competitive performance against state-of-the-art methods and yields only a slight improvement over DigNet under comparable conditions. Compared with conventional diffusion models that rely on fixed sampling schedules, Planet employs a fast-sampling strategy that accelerates inference with only minimal accuracy trade-off. When applied to mouse-lung Cd8^+^Gzmk^+^ T cells, Planet successfully reconstructs a cell-type-specific GRN, recovers both established and previously uncharacterized regulators, and delineates the dynamic immunoregulatory changes that accompany ageing. Overall, Planet provides a practical framework for constructing cell-specific GRNs with improved global consistency, offering a complementary perspective to existing methods and new insights into regulatory dynamics in health and disease.

## 1. Introduction

Deciphering the gene regulatory networks (GRN) that orchestrate cell fate decisions and mediate cellular adaptation to environmental cues remains one of the most fundamental and enduring challenges in systems biology [1]. With the explosive growth of high-throughput sequencing technologies, particularly single-cell RNA sequencing (scRNA-seq), the volume and dimensionality of transcriptomic data have increased exponentially [2]. These data offer unprecedented opportunities to reconstruct GRN at single-cell resolution through reverse engineering approaches. Despite this progress, robustly and generalizably inferring regulatory interactions remains an unresolved problem [3]. True regulatory links are typically sparse, condition-dependent, and highly nonlinear, while transcriptomic measurements are frequently corrupted by noise and batch effects [4,5,6]. Consequently, reconstructing biologically meaningful GRN with global consistency from sparse and noisy data continues to be a formidable task at the interface of computational biology and medicine.

A wide spectrum of computational approaches has been proposed to address this challenge. Classical statistical and machine learning methods, including correlation-based metrics, probabilistic graphical models, and differential equation-based algorithms, have achieved success on specific benchmarks but often falter when confronted with noisy single-cell RNA sequencing (scRNA-seq) data or complex regulatory architectures. Representative algorithms such as GENIE3 [7], SCENIC [8], and SCODE [9] demonstrate strengths in certain contexts, yet suffer from limited noise tolerance, strong prior assumptions, and the need to infer regulatory edges in a pairwise manner. More recent deep learning–based strategies, including GENELink [10] and CNNC [11], exploit attention mechanisms and high-dimensional representation learning to uncover complex dependencies. However, their reliance on supervised training with prior knowledge constrains their general applicability. Unsupervised generative frameworks such as DeepSEM [12] alleviate this limitation by leveraging variational autoencoders to reconstruct latent regulatory structures, but their performance often depends critically on dataset size and may degrade when scaling to genome-wide networks. Recent frameworks, including DeepMAPS [13], CellOracle [14], Dictys [15], and LINGER [16], integrate epigenomic features to improve prediction reliability and biological interpretability. However, their applicability is constrained by the requirement for multi-omics inputs, making them inoperable when only transcriptomic data are available. Although multi-omics data provide richer biological information, they are more difficult to acquire and therefore have a narrower range of practical applicability.

In parallel, the emergence of deep generative models, particularly denoising diffusion probabilistic models (DDPM) [17], has fundamentally reshaped the way complex high-dimensional data distributions can be modeled. Diffusion models learn to reverse a progressive noise-injection process, thereby enabling the generation of structured outputs such as networks, images, and sequences in an unsupervised or semi-supervised fashion [18,19], so they are also suitable for GRN reverse engineering tasks. Compared with continuous Gaussian formulations, discrete diffusion frameworks better preserve sparsity and graph topologies. When augmented with conditional embeddings, these models can further achieve controllable generation of structured biological networks [20]. Building on this intuition, DigNet [21] recently framed GRN inference as a discrete diffusion reversal problem on scRNA-seq data, achieving cell-specific regulatory networks and improved global consistency over conventional one-shot inference. Nonetheless, DigNet presents two critical limitations: (i) its attention mechanism is restricted to node-level features, precluding integration of hierarchical or cross-level information, and (ii) structural feedback from intermediate diffusion steps is underexploited, resulting in incomplete retention of network topology.

To address these challenges, we present Planet, an attention-guided probabilistic diffusion framework for generating GRN. Planet is founded on a discrete diffusion process coupled with a hybrid attention architecture that integrates three complementary mechanisms: a graph attention module for dynamic feature aggregation, a cross-attention mechanism for structural memory alignment across time steps, and multi-head self-attention to capture diverse regulatory patterns. This design enables the model to concentrate on biologically salient features, preserve high-confidence edges, and incorporate temporal information across the diffusion trajectory. In addition, we introduce an early-stopping diffusion strategy that prunes redundant edges, thereby accelerating network generation without sacrificing accuracy. Extensive evaluations across both simulated and benchmark transcriptomic datasets, including breast cancer data, demonstrate that Planet consistently surpasses state-of-the-art methods such as DigNet in AUROC and AUPRC while achieving substantially faster generation. Beyond these benchmarks, we further applied Planet to mouse lung Cd8^+^Gzmk^+^ T cells, where it successfully generated cell type–specific GRN, recovered both established and previously uncharacterized regulators, and delineated dynamic immune regulatory changes associated with aging. Collectively, these results highlight the potential of combining diffusion-based generative modeling with hybrid attention as a scalable and biologically interpretable paradigm for GRN generation, with broad implications for systems biology and precision medicine. The contributions of this work can be summarized as follows:

- Hybrid multi-level attention for complex regulation modeling. Planet integrates graph attention for structure-aware local aggregation, cross-attention for preserving global regulatory architecture across diffusion steps, and multi-head self-attention for capturing long-range dependencies. Together, these preserve high-confidence edges, enable structure-guided feature aggregation, and dynamically modulate information across diffusion steps, allowing stable modeling of complex network under high noise and sparsity.
- Dynamic exploitation of time-step information. By means of time embeddings, it maps gene-expression and regulatory features into a shared temporal space: global structure is retained at high-noise steps, local details are emphasized at low-noise steps, and node- and edge-specific signals are fed back into the network structure, improving robustness and biological consistency.
- Efficient diffusion with accelerated sampling. With the training objective unchanged, accelerated sampling achieves generation quality comparable to full-step sampling while using fewer reverse steps, reducing computation and latency and offering a practical path for large-scale network and multi-condition scenarios.

## 2. Materials and Methods

### 2.1. Planet Framework

**Figure 1:**
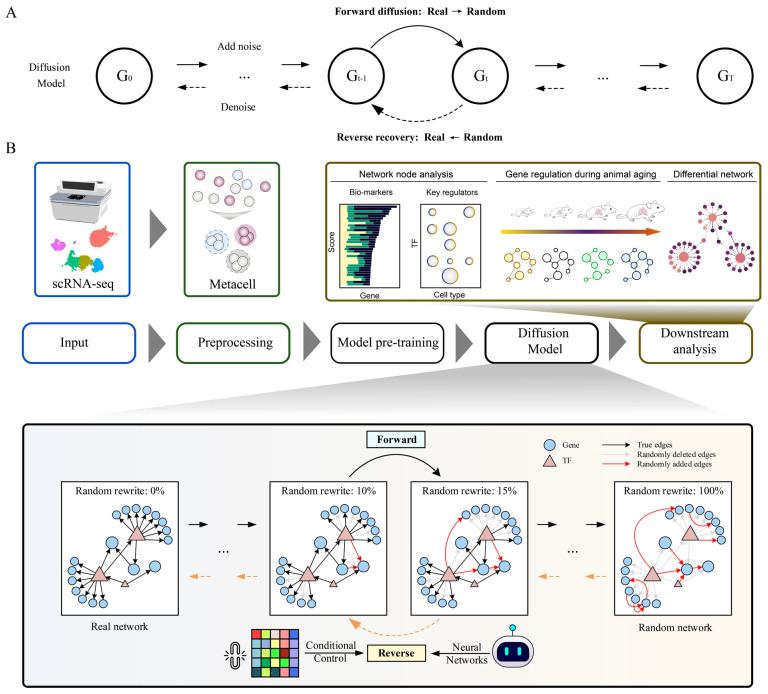
Overview of the diffusion paradigm and the Planet framework. (**A**) Conceptual schematic of a generic diffusion model; (**B**). Workflow of the Planet model for generating a GRN from gene ex-pression profiles. The process comprises five main stages. Step 3 (model pre-training) is optional when pre-trained weights are available. Step 4 (the diffusion module) serves as the core component, where a conditional controller, such as a gene expression profile, guides the generative trajectory through a pre-trained neural network to reconstruct the GRN.

Diffusion-based generative models have demonstrated remarkable success in domains such as image synthesis and molecular design (Figure 1A). However, their application to GRN inference, a central problem in systems biology, remains largely unexplored. GRN capture the complex regulatory interactions that govern gene expression and underlie cellular identity, plasticity, and disease progression. Accurate reconstruction of GRN is therefore critical for advancing our understanding of dynamic regulatory mechanisms, yet existing approaches often suffer from noise sensitivity, loss of structural consistency, and limited interpretability.

To address these challenges, we present Planet, a discrete diffusion model augmented with a hybrid attention mechanism that directly generates GRN from gene expression profiles (Figure 1B). Unlike conventional approaches that treat each diffusion step independently, Planet integrates information across the generative trajectory. In the early diffusion stages, a GATv2 module adaptively balances feedback from the preceding step with the original expression profiles, enabling dynamic denoising. At subsequent steps, self-attention–derived structural proposals are reconciled with partially inferred networks through cross-attention, which highlights and preserves high-confidence regulatory interactions. Finally, a feature-pooling module consolidates both gene-level and regulatory features into time-aware embeddings, aligning nodes and edges with diffusion steps and capturing temporal dynamics with high fidelity. Planet is a general and extensible GRN generation framework that requires only gene expression profiles as input. It can therefore be readily applied to any condition, sample, or organism, including other human single-cell datasets with similar data structure.

Collectively, this architecture endows Planet with the ability to maintain structural coherence, improve robustness to stochastic noise, and recover biologically plausible networks with enhanced interpretability. Beyond accurate GRN reconstruction, the generated networks enable downstream analyses such as node centrality assessment, differential network comparison, and identification of dynamic regulatory mechanisms, thereby providing a new tool for dissecting transcriptional regulation in complex biological systems.

### 2.2. Diffusion Model Framework

#### 2.2.1. Framework Overview

Planet uses a diffusion-based architecture to model nonlinear regulatory relationships among genes while conditioning on the cellular state defined by observed gene expression, thereby linking expression profiles to the structure of the GRN. Specifically, Planet applies a discrete diffusion process to progressively recover the correct regulatory edges from a network initialized from a preset random distribution. We denote the GRN as G(V, E) with node set V and edge set E. Nodes include transcription factors and genes, and edges are directed from transcription factors to their target genes. Each edge in *E* is binary: present (1) or absent (0). Starting from a random graph G, Planet generates a GRN that matches the input expression profile and, via an ensemble module, returns an edge-level probability. The model has two components: a forward diffusion process and a reverse denoising process. The forward diffusion, used only during training, is parameter-free and serves to learn from large collections of networks. The reverse denoising process is used during both training and testing; it is a parameterized neural network trained to iteratively transform a random network into the target network.

#### 2.2.2. Forward Process

We define a forward diffusion process that progressively corrupts the target network $E^{0}$. At each time-step t, Gaussian noise is added and random sampling produces a perturbed network $E^{t}$.

(1) $$q\left(E^{1},\ldots ,E^{T}|E^{0}\right)=q\left(E^{1}|E^{0}\right)\prod_{t=2}^{T}q\left(E^{t}|E^{t-1}\right).$$

Following prior work, the noise between diffusion steps is represented by a state transition matrix M:

(2) $$M^{t}=\alpha^{t}S+\left(1-\alpha^{t}\right)I,$$

where S is the noise distribution matrix that encodes the ratio between true edges in the GRN and all possible edges. Following prior work, we use a cosine noise schedule since the optimal α for GRN remains unknown. In addition, by specifying S, the model gradually drives M from an initial matrix $\left[\begin{array}{cc} 1 & 0\\ 0 & 1 \end{array}\right]$ to $\left[\begin{array}{cc} \delta & 1-\delta \\ \delta & 1-\delta \end{array}\right]$. This transition matrix enforces network sparsity, aligning random network with the prior GRN structure. Moreover, the matrix admits a closed-form solution, allowing the corrupted network after t steps to be written explicitly rather than computed recursively:

(3) $$q\left(E^{t}\right)=E^{t-1}M^{t}=E^{0}\prod_{i=2}^{t}M^{i}=E^{0}{\bar{M}}^{t}.$$

Thus, at any given time, the noisy network can be obtained from the initial network $E^{0}$ and a fixed noise level ${\bar{M}}^{t}$. Clearly, Equation (3) alone cannot guarantee network sparsity. To ensure both sparsity and discretization, we additionally apply simple random sampling, namely:

(4) $$q(E^{t}|E^{t-1})=Discrete(E^{t};\pi =E^{t-1}M^{t}),$$

(5) $$q(E^{t}|E^{0})=Discrete(E^{t};\pi =E^{t-1}{\bar{M}}^{t}).$$

Discrete(.) denotes the simple random sampling process, and π represents the sampling probability. Since the noise matrix M is a fixed hyperparameter, the network $E^{t}$ at any time-step can be derived directly from the initial state $E^{0}$.

#### 2.2.3. Reverse Process

The reverse process restores $E^{t}$ to $E^{t-1}$ by progressively removing noise from the network. During reverse sampling, we adopt a DDIM-style procedure that significantly accelerates inference while maintaining generation quality. Given a sparse set of inference time-steps $\tau =\left[0,\tau_{1},\ldots ,\tau_{k}\right]$ with k<T, sampling starts from $E^{\tau_{k}}$ and ends at $E^{\tau_{k}}$. This strategy greatly reduces the number of sampling steps (e.g., from 1000 to 500 or fewer) while preserving high quality, as shown by Song et al. [22]. By Bayes’ theorem and the Markov property,

(6) $$q(E^{\tau_{k-1}}|E^{\tau_{k}},E^{0})=\frac{q(E^{\tau_{k}}|E^{\tau_{k-1}},E^{0})q(E^{\tau_{k-1}},E^{0})}{q(E^{\tau_{k}}|E^{0})},$$

It suffices to recover $E^{\tau_{k-1}}$ from $E^{\tau_{k}}$ if $E^{0}$ already exists. Since $E^{0}$ is unknown, we cannot directly evaluate $q(E^{\tau_{k-1}}|E^{\tau_{k}},E^{0})$. We therefore introduce a custom deep neural network, TriHAT (Triple Hybrid-Attention Transformer), to produce an estimate $\varphi_{\theta }(E^{0})$. Based on this model, the denoised network can be obtained via $q(E^{\tau_{k-1}}|E^{\tau_{k}},\varphi_{\theta }(E^{0}))$. Although predicting $\varphi_{\theta }(E^{0})$ with a neural network and then deriving $E^{\tau_{k-1}}$ is more involved, it stabilizes training and provides a clear optimization direction. The equations used to compute $E^{\tau_{k-1}}$ via the neural network are summarized as follows:

(7) $$p_{\theta }(E^{\tau_{k-1}}|E^{\tau_{k}})\propto q(E^{\tau_{k}}|E^{\tau_{k-1}},\varphi_{\theta }\left(E^{0}\right))p_{\theta }(\varphi_{\theta }\left(E^{0}\right)|E^{\tau_{k}}).$$

To train the neural network $\varphi_{\theta }(E^{0})$, we define the loss as the cross-entropy between the predicted network $\varphi_{\theta }(E^{0})$ and the ground-truth network $E^{0}$, i.e., $cross-enropy\;(\varphi_{\theta }(E^{0}),E^{0})$. This objective directly focuses the model on learning the true network structure, without auxiliary tasks. Once the model is trained, generation reduces to running the reverse process: progressively denoising from t=k down to t=0.

### 2.3. Triple Hybrid-Attention Transformer for Generative GRN

The reverse process requires a neural network to proceed effectively, and for this purpose we designed TriHAT (Triple Hybrid-Attention Transformer), a model that integrates three distinct forms of attention mechanisms. It consists of two main components: a time-step-guided Graph Attention Encoder (GAT) for capturing local topological relationships and modeling structured interactions between nodes, and a Graph Transformer Encoder that, through multiple layers of self-attention and cross-attention modules, integrates node, edge, and temporal information. The TriHAT first refines node features, which are then processed by the Graph Transformer to perform joint graph representation learning and predict the reverse diffusion trajectory. By combining graph, cross-, and self-attention, TriHAT achieves robust and interpretable GRN generation under noise and sparsity. The following describes the GAT and Graph Transformer in detail.

#### 2.3.1. Graph Attention Mechanism

To aggregate features from the previously feedback GRN and refine node feature representations, we introduce a two-layer multi-head GATv2 module [23]. GATv2 demonstrates improved numerical stability and generalization when deeply stacked, making it suitable for modeling complex regulatory network. The attention mechanism for each layer is configured as follows:

(8) $$e_{ij}=a^{T}LeakyReLU\left(W\left[h_{i}||h_{j}\right]\right),$$

(9) $$\alpha_{ij}={softmax}_{j}(e\left(h_{i},h_{j}\right)),$$

(10) $$h_{i}^{\prime }=\sum_{j\in N_{i}}\alpha_{ij}Wh_{j},$$

where $h_{i}$ denotes the node feature vector, W is a learnable linear mapping, and α is the attention vector. We configure GATv2 with multiple attention heads and aggregate the outputs of different heads either by averaging or concatenation, to learn feature parameters from different semantic subspaces. In addition, this module introduces a temporal encoding layer that maps the current time-step into an embedding for computing attention coefficients.

By combining these time-encoded attention coefficients with the gene embeddings, the updated node feature representation is expressed as follows:

(11) $${\tilde{h}}_{i}=(1-\sigma \left(t\right))\cdot h_{i}^{\prime }+\sigma \left(t\right)\cdot x_{i},$$

where σ denotes the activation function in the linear layer. The final node features are then obtained by concatenating the original input data with the learned features, followed by a mapping operation to produce the output representation:

(12) $${\tilde{x}}_{i}=\sigma (\left[{\tilde{h}}_{i}||x_{i}\right]).$$

In the diffusion model, introducing time-step–based feature fusion allows the structural information to retain a global outline in high-noise stages while emphasizing local details in low-noise stages, thereby helping stabilize the reverse diffusion process.

#### 2.3.2. Graph Transformer Encoder

**Figure 2:**
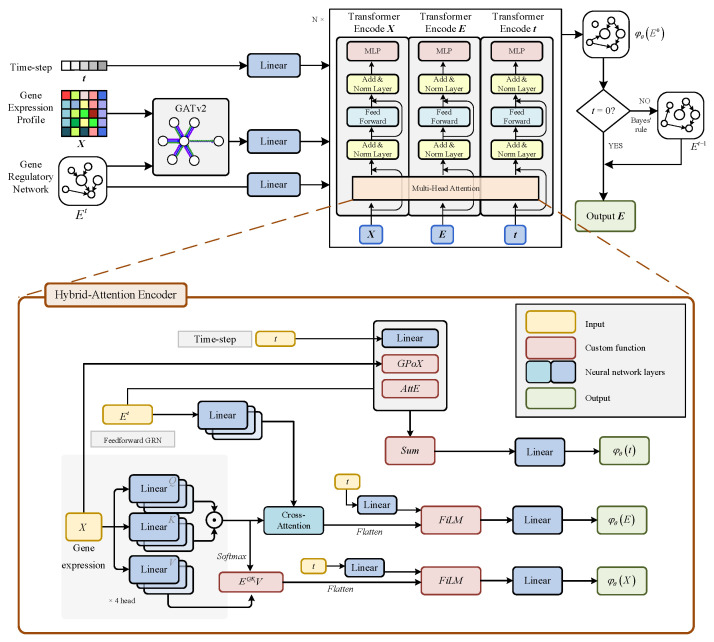
Triple hybrid-attention transformer architecture.

As shown in Figure 2, the multi-layer graph transformer encoder [20] is an improved version of the traditional transformer architecture and is designed to update three variables: X (gene embeddings), E (edge embeddings), and t (time-step embeddings). Among these, *X* is updated and optimized through a multi-head self-attention mechanism, computed as follows:

(13) $$Attention\left(X\right)=Softmax\left(\frac{QK^{T}}{\sqrt{d_{k}}}\right)V=Softmax\left(\frac{{lin}_{Q}\left(X\right){\left({lin}_{K}\left(X\right)\right)}^{T}}{\sqrt{d_{k}}}\right){lin}_{V}\left(X\right),$$

where Q, K, and V are obtained by mapping the gene embeddings through separate linear layers.

The attention-weighted gene embeddings are then fused with the time information via *FiLM* [24] function as follows:

(14) $$\hat{X}=FiLM(t,Attention\left(X\right)),$$

(15) $$FiLM\left(a,b\right)={lin}_{1}\left(a\right)+{lin}_{2}\left(a\right)\cdot b+b.$$

The edge embedding must be updated and optimized by combining self-attention and cross-attention mechanisms. Specifically, the self-attention mechanism is first applied to obtain gene regulation relationships guided by gene expression:

(16) $$E^{self}=\frac{{lin}_{Q}\left(X\right){\left({lin}_{K}\left(X\right)\right)}^{T}}{\sqrt{d_{k}}}.$$

Then, a cross-attention mechanism CrossA(·) is introduced to fuse the feed-forward GRN $E^{feed}$ with the GRN $E^{self}$ generated by multi-head self-attention. In this process, TriGAT applies separate linear layers to process $E^{feed}$ (feed-forward) and $E^{self}$ (multi-head self-attention output). $E^{feed}$ is obtained by integrating the feed-forward network with the time-step information via $E^{feed}=FiLM(y,E^{feed})$ fusion. Finally, letting ${lin}_{Q}(E^{self})$ be Q, ${lin}_{K}(E^{feed})$ be K, and ${lin}_{V}(E^{feed})$ be V, the final edge embeddings $\hat{E}$ are computed using the following attention equation:

(17) $$\hat{E}=CrossA(E^{self},E^{feed})=Softmax\left(\frac{{lin}_{Q}(E^{self}){\left({lin}_{K}(E^{feed})\right)}^{T}}{\sqrt{d_{k}}}\right){lin}_{V}(E^{feed}),$$

This module uses $E^{self}$ as the core representation, refining certain regulatory relationships in $E^{self}$ with information from $E^{feed}$, while reinforcing and preserving the knowledge learned in earlier stages.

To update the time-step embedding t, both gene information and regulatory information are taken into account, and separate processing functions are designed for each. Gene features X are first processed through a Sigmoid gating mechanism combined with pooling, then mapped into the time-step space via a linear layer. In parallel, a custom self-attention mechanism is applied to pool the edges in the feed-forward network E, and the pooled edge features are also mapped into the time-step space through a linear layer. By combining these two mapped feature sets with the original time embedding, the resulting time-step embedding incorporates multi-level information:

(18) $$\hat{t}=Linear\left(t\right)+GPoX\left(\hat{X}\right)+AttE\left(\hat{X},\hat{E}\right),$$

(19) $$GPoX\left(x\right)=W_{lin}^{T}\left(\sum_{i=1}^{n}{\left(\sigma \left(W_{g}x+b_{g}\right)⊙x\right)}_{i,*}\right)+b_{lin},$$

where the GPoX layer and AttE layer perform feature pooling on X and E respectively.

Overall, based on Equations (13), (15) and (17), each layer of the Graph Transformer updates $\hat{X}$, $\hat{E}$, and $\hat{t}$. The designed multi-layer graph transformer architecture enables the model to more deeply analyze data distribution patterns and more accurately characterize the concordance between gene expression and gene regulatory links.

### 2.4. Benchmark Datasets and Preprocessing

To rigorously evaluate Planet and competing GRN inference models, we employed both simulated and experimental single-cell transcriptomic datasets.

#### 2.4.1. Simulated Datasets

We first used the SERGIO platform [25] to construct benchmark GRN and generate corresponding single-cell gene expression profiles. During GRN design, the number of gene nodes was randomly sampled from a uniform distribution and subsequently partitioned into transcription factors (TF) and non-TF genes. Network generation followed three biologically motivated constraints: (1) the number of edges was restricted to between n and 2n to preserve realistic sparsity; (2) self-regulatory edges were excluded; and (3) TFs regulated target genes in a strictly unidirectional manner. Based on these criteria, we simulated 150 GRN, of which 100 were used for training and 50 were reserved as an independent test set. For each GRN, expression matrices were generated via SERGIO built-in stochastic differential equation model, yielding 100 simulated single-cell samples per network. This setting provides a controlled environment for benchmarking and enables systematic comparison of model performance under varying network topologies.

#### 2.4.2. Breast Cancer Dataset

To assess performance on experimental data, we incorporated a breast cancer scRNA-seq dataset reported by Qian et al. (EMBL-EBI ArrayExpress accession: E-MTAB-8107) [26]. The dataset contains annotated cell types and underwent stringent quality control: samples with fewer than 100 cells and cells or genes with >95% missing values were excluded. After preprocessing, the dataset comprised 11,331 cells spanning eight major cell types across five patient samples. Cell-type abundances ranged from 148 to 2938, and included T cells, B cells, myeloid cells, tumor cells, dendritic cells, endothelial cells, fibroblasts, and mast cells. To mitigate dropout effects intrinsic to scRNA-seq, we applied the SAVER algorithm [27] for matrix completion, followed by iMetacell [21] aggregation to enhance feature robustness. This dataset allows Planet to be evaluated in the context of heterogeneous tumor microenvironments, where accurate GRN inference can provide insight into cell-type–specific regulatory programs.

#### 2.4.3. Mouse Aging Dataset

To investigate transcriptional regulation in aging, we analyzed the mouse single-cell aging atlas generated by Zhang et al. [28]. This atlas profiles multiple organs and cell types, from which we extracted Cd8^+^Gzmk^+^ T cells from the lung, as annotated in the original study. The dataset encompasses five age groups (3, 6, 12, 16, and 23 months) representing key stages of the murine lifespan, with cell counts per group ranging from 183 to 6804. Prior analyses by Zhang et al. revealed both shared and age-specific features in this population. By applying Planet to reconstruct cell-type–specific GRN across these age groups, we aimed to uncover molecular drivers of age-associated regulatory changes. To account for the high sparsity of single-cell data, iMetacell [21]. was again applied to aggregate features, thereby improving the stability and interpretability of inferred networks.

Together, this combination of simulated, cancer-derived, and aging-related datasets provides a comprehensive benchmark, enabling evaluation of Planet across both controlled and biologically complex contexts.

## 3. Results and Discussion

### 3.1. Synthetic Benchmarking Confirms Planet Efficacy

**Figure 3:**
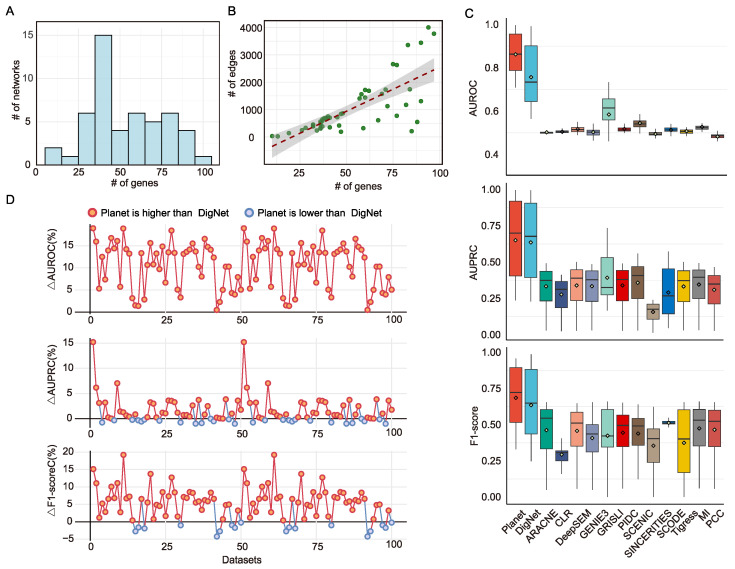
Benchmarking on simulation datasets. (**A**) Distribution of gene counts in the simulated datasets; (**B**) Ratio of genes to regulatory interactions in each simulated dataset; (**C**) Performance comparison of 14 methods on simulated datasets using three evaluation metrics; (**D**) Detailed comparative analysis between Planet and DigNet.

Synthetic datasets provide a rigorous and controlled basis for assessing GRN generation models, as they enable systematic evaluation against networks with known ground truth. To this end, we adopted the DigNet simulation framework and employed SERGIO to generate 100 training sets and 50 independent test sets, thereby ensuring both generalization and statistical reliability. The distributions of genes and edges across synthetic networks are summarized in Figure 3A,B.

We benchmarked Planet against 13 widely used GRN inference methods, including ARACNE [29], CLR [30], DigNet [21], DeepSEM [12], GENIE3 [7], GRISLI [31], PIDC [32], SCENIC [8], SCODE [9], SINCERITIES [33], Tigress [34], Mutual information (MI) and PCC (Pearson correlation coefficient), with detailed configurations listed in Table 1. For fairness, all models were executed under optimal or author-recommended hyperparameters and repeatedly evaluated across the 50 test sets. Planet and DigNet were trained under identical hyperparameter settings, including learning rate, optimizer, training epochs, and network size. The same preprocessed datasets were used for both methods. Where hyperparameters were model-specific, we followed each method’s original recommendations. Performance was assessed by AUROC, AUPRC, and F1-score (Figure 3C). Planet achieved substantial gains over all baselines: compared to the next-best model, DigNet, AUROC improved by 10.52% (32.25% average relative increase), AUPRC by 1.50% (30.58% average relative increase), and F1-score by 5.04% (24.57% average relative increase).

Per-dataset analysis further demonstrated that Planet consistently outperformed DigNet across networks of varying sizes, yielding higher AUROC on the vast majority of test sets (Figure 3D). This robustness indicates that the hybrid attention design more effectively captures both local and global structural information, without bias toward network density or scale. Importantly, Planet not only surpasses state-of-the-art GRN methods in predictive accuracy but also reduces computation time and improves efficiency, owing to its early-stopping diffusion strategy. Together, these results establish Planet as a practical and scalable generative framework for GRN construction, validating the advantages of coupling diffusion modeling with multi-level attention in synthetic benchmarks.

### 3.2. Accelerated Sampling Strategy and Module Contributions

Diffusion-based generative models are often computationally expensive, so Planet introduces an accelerated sampling strategy. When training with a schedule of length T, the learned objective effectively covers any subsequence of time steps. For any subsequence $\left[\tau_{0},\ldots ,\tau_{k}\right]\subset \left[0,\ldots ,T\right]$, we define objectives $L_{T}$ and $L_{\tau }$, with $L_{\tau }$ being a subset of $L_{T}$. When training is sufficiently converged, optimizing $L_{T}$ approximates optimizing $L_{\tau }$, thus a model trained under $L_{T}$ can be used with a reduced sampling schedule of k steps. In practice, we keep the training procedure unchanged and shorten the sampling path from the original t=T→0 to t=k→0.

**Figure 4:**
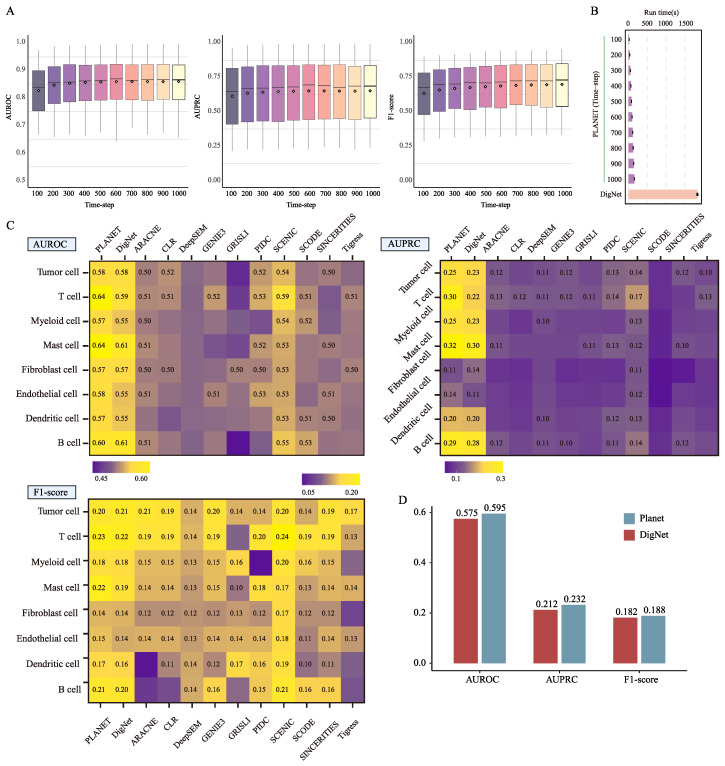
Model efficiency and comparative performance evaluation. (**A**) Efficiency of Planet under different time-step acceleration settings, assessed by three evaluation metrics; (**B**) Analysis of runtime; (**C**) Comparative performance of 12 state-of-the-art algorithms on the breast cancer dataset, evaluated across the same three metrics; (**D**) Performance comparison of Planet and DigNet in (**C**) (average).

On 50 simulation datasets, after training for 1000 time-steps, we froze the parameters and evaluated sampling performance for schedules from 100 to 1000 steps at intervals of 100 (Figure 4A,B). Performance improves as the number of steps increases and stabilizes beyond 500 steps. A streamlined network design further speeds inference by avoiding redundant computation. Even without reducing the sampling steps, Planet model sampling time is markedly shorter than DigNet model, improving computational efficiency; accelerated sampling offers an even faster option.

The key modules of Planet model are GATv2 module, the cross-attention module, and the feature-pooling module, responsible for initial feature aggregation, prior-structure integration, and multi-feature fusion (gene expression, gene regulation, and temporal signals), respectively. We ablated these components by removing or replacing them and tested their contributions on 10 simulated datasets (Table 2). The full Planet model achieved an AUROC of 0.9136 and an AUPRC of 0.8063. Relative to this baseline, the three modules contribute similarly, yielding gains of 0.7%~0.9% (AUROC) and 2.0%~2.2% (AUPRC). Removing both GATv2 and feature pooling had the largest impact, reducing performance by 1.4% and 2.6% on the two metrics, respectively.

### 3.3. Planet Generates Reliable Network on Breast Cancer Data

Next, we evaluated Planet on the breast-cancer single-cell dataset curated by Qian et al. [26] which comprises eight cell types from five patients. In real-world single-cell data, the absence of a gold-standard regulatory network makes reliable training and performance assessment challenging. Following the strategy of Wang et al. [21], we constructed cell-type-specific reference networks for each cell type by aggregating interactions from RegNetwork databases and augmenting them with highly correlated edges. These references are used both to training and to evaluate the reliability of the generated networks. Given the limited amount of training data and the high complexity of hole-genome GRNs, we decomposed each full network into multiple subnetworks, and the gene sets in each subnetwork are consistent with the pathway modules in the KEGG database to ensure biological interpretability. We then designated the subnetwork corresponding to the breast cancer pathway (KEGG: hsa05224) together with its associated expression profiles as the held-out test set, while subnetworks derived from all other pathways are used for model training. To ensure a fair evaluation, any gene overlapping with the breast-cancer pathway was removed from the training set, yielding a strict separation between training and test genes.

As shown in Figure 4C,D, Planet generally outperforms competing methods across metrics, with the strongest gains on AUROC, averaging a 2~14% improvement over advanced baselines. For AUPRC, Planet ranks first for most cell types and averages a 2~18% improvement, except in Fibroblast cells where it is second to DigNet. The AUROC and AUPRC results indicate that Planet estimates regulatory edge probabilities effectively. For F1-score, Planet is slightly below SCENIC and ranks second, suggesting that, after thresholding, its precision–recall balance is marginally less favorable than SCENIC’s. Compared with the overall second-best method (DigNet), Planet improves AUROC, AUPRC, and F1-score by approximately 2%, 2%, and 0.7% on average across cell types. This low variance may be due to technical noise in the data and missing annotations in the reference network. Performance is strongest in T cell–specific GRN and weakest in myeloid cell–specific GRN. In terms of overall performance, Planet performs only slightly better than DigNet. In terms of overall performance, Planet performs only slightly better than DigNet. Additional comparative evaluations between the two methods are provided in the Supplementary Materials (Supplementary Note S1, Tables S1 and S2, and Figure S1).

### 3.4. Planet Reveals Regulatory Mechanisms in Mouse Aging

**Figure 5:**
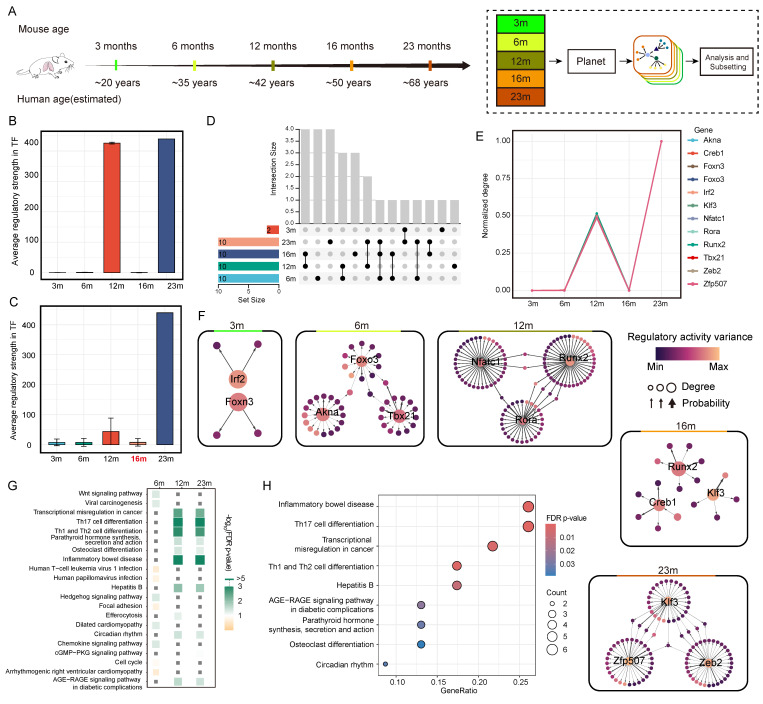
Regulatory mechanisms during mouse aging. (**A**) Illustration of applying Planet to mouse aging data. (**B**) Summary of TF regulatory activity from cell-type-specific GRN con-structed by Planet. (**C**) Summary of TF regulatory activity from cell-type-specific GRN con-structed by DigNet. (**D**) Overlap of the top 10 TFs in each cell-type-specific GRN across aging stages. (**E**) Normalized regulatory activity of the top three TFs in each GRN across aging stages. (**F**) Cell-type-specific GRN generated by Planet, showing the top three TFs and their associated regulatory edges for each stage. (**G**) Summary of KEGG pathway enrichment analysis of the top 50 variable genes from GRN at 6, 12, and 23 months. (**H**) Detailed KEGG enrichment analysis of key genes from the 12-month GRN with visualization.

Following Zhang et al., we focus on Cd8^+^Gzmk^+^ T cells in the lung to enable cell type–specific analysis [28]. As aging progresses, Zhang et al. reported aberrant expansion of activated Cd8^+^Gzmk^+^ T cells across multiple organs, with both the abundance and expression of Granzyme K elevated in aged mice, findings consistent with prior studies [35]. While simple statistical summaries can provide a rough view of changes in cell abundance or gene expression across organs and time points, they obscure the contribution of internal molecular regulation to the aging process. We therefore analyzed Cd8^+^Gzmk^+^ T cells across time points to resolve the key regulatory mechanisms underlying aging (Figure 5A). Before constructing cell-specific GRN, we defined a focused gene set to streamline the analysis and improve computational efficiency.

Using the cell-specific GRN constructed by Planet, we analyze the transcription factor (TF) regulatory intensity in Cd8^+^Gzmk^+^ T cells from the lungs of mice at different ages. We find that the average regulatory intensity at 12 months and 23 months is significantly higher compared to other time points (Figure 5B). At 12 months, the regulatory intensity rises sharply, reaching its peak at 23 months, while the 3, 6, and 16 months exhibit low levels close to baseline. This result suggests a marked increase in transcriptional regulation of immune cells during mid-age (12 months), with a sustained rise continuing into old age (23 months). This was also confirmed by the results of the DigNet algorithm (Figure 5C). To further confirm this pattern, we extract hub TFs from the specific GRN for each time point and examine the overlap among the top 10 TFs across stages (Figure 5D). At 3m, only two hub TFs are detected, with minimal overlap with other stages, suggesting low transcriptional regulatory activity in Cd8^+^Gzmk^+^ T cells of young mice. In contrast, there is substantial overlap among the 12, 16, and 23 months, with TF composition stabilizing after 12 months and persisting through 23 months. These findings suggest that certain core TFs are activated in midlife and remain engaged in gene regulation during aging. Although the inferred GRNs provide valuable hypotheses regarding transcription factor regulation, these findings should be interpreted cautiously, as they rely on computational inference and have not yet been experimentally confirmed.

We further compare the regulatory intensities of the top 3 key TF at each stage (Figure 5E). Nearly all TF included in the analysis, such as Foxo3, Klf3, Tbx21, Nfatc1, Zeb2, and Creb1, show an upward trend at 12 months, a brief decline at 16 months, and a second peak at 23 months. This indicates that the transcriptional regulatory network is highly active at both 12 and 23 months. Previous studies report that TF including Foxo3, Klf3, and Tbx21 are involved in T cell activation, inflammatory responses, and enhancement of effector functions, and that their expression and activity can change dynamically during aging [35,36,37]. Creb1 has been identified as a key regulator in the aging process [38]. In addition, Nfatc1 and Zeb2 have been shown to drive terminal differentiation of effector T cells and influence immune aging–related phenotypes [39,40]. Our results are consistent with these findings, suggesting that immune cell transcriptional networks undergo stage-specific enhancement during mid-life and old age, closely linked to age-related changes in immune regulation.

From the constructed cell-specific GRN, we extract the downstream subnetworks corresponding to 12 TFs (Figure 5F). In the figure, node size represents the out-degree of each gene (i.e., the number of downstream genes it regulates), while node color reflects the variance in its total regulatory intensity across time points, providing an indication of its dynamic regulatory activity. Edge thickness denotes the confidence probability of each regulatory link. To enhance network readability, low-confidence connections are removed. At the key time point of 12 months, we observe a high-probability regulatory link from Runx2 to Smad3, which aligns closely with previous reports on the Runx2 → Smad3 axis in signal transduction and development [41]. Notably, the regulatory intensity variance in Smad3 is as high as 27.31, ranking second among all candidate genes, suggesting that it may play a significant regulatory role across different time points. Additionally, we identified several potentially biologically significant but experimentally unverified regulatory paths, such as Nfatc1 regulating Ruvbl2 and Rora regulating Nek7. Although existing research highlights the key roles of these genes in aging-related mechanisms [42,43], the specific transcriptional regulatory relationships still require further experimental validation.

To systematically explore potential functional reprogramming of lung T cells during aging, we use the cell-specific GRN constructed for three key time points (6, 12, and 23 months, consistent with the grouping of Zhang et al.) and select the top 50 genes with the highest variance in each network for KEGG pathway enrichment analysis (Figure 5G). Overall, most pathways show marked activation at 12 months, followed by either maintenance, decline, or further enhancement at 23 months, revealing a “mid-life activation–late-stage stabilization/restructuring” trajectory of transcriptional function. Immune-regulation–related pathways, including Th17 and Th1/Th2 cell differentiation, transcriptional misregulation in cancer, and the AGE–RAGE signaling pathway, peak in enrichment at 12 months, suggesting a pronounced immune fate shift and enhanced inflammatory regulation in T cells during mid-life [44,45]. The detailed enrichment profile for 12 months is shown in Figure 5H. At this stage, we also observe activation of noncanonical immune pathways such as circadian rhythm, indicating that T cells enter a state of systemic reprogramming associated with aging. This collective activation of transcriptional network suggests that 12 months is a pivotal transition point for immune fate shift, pro-inflammatory phenotype remodeling, and disruption of immune homeostasis. By 23 months, although some pathways, such as Th17 differentiation and AGE-RAGE signaling, remain highly enriched, the overall enrichment intensity stabilizes, possibly reflecting a state of functional exhaustion or immune suppression in T cells driven by long-term inflammation.

## 4. Conclusions

In this study, we introduce Planet, a diffusion-based generative framework for reconstructing GRN directly from gene expression data. By incorporating a hybrid-attention mechanism, Planet integrates information across diffusion time steps, thereby capturing both global network architecture and fine-grained local interactions with high fidelity. This design ensures structural consistency throughout the generative process while enhancing robustness to noise and sparsity, two major challenges in single-cell transcriptomics. Benchmarking with state-of-the-art GRN inference methods indicates that Planet delivers consistent and biologically meaningful results, with performance comparable to or slightly exceeding existing approaches. Its ability to leverage time-step information and support accelerated sampling further makes it a scalable and practical solution for large-scale or multi-condition analyses. Beyond methodological advances, application of Planet to mouse lung Cd8^+^Gzmk^+^ T cells uncovered stage-specific transcriptional programs underlying immune aging, revealing regulatory modules that both corroborate and extend previous biological knowledge.

Taken together, these findings suggest that Planet represents a practical and extensible framework for single-cell GRN inference, offering complementary insights to existing methods. By bridging methodological innovation with biological discovery, Planet not only advances the field of computational regulatory genomics but also provides a foundation for exploring dynamic cellular processes in development, disease, and aging. In addition to these applications, Planet can be readily extended to other use cases, including tumor microenvironment analysis, cell reprogramming, and cross-species regulatory network comparison. Its diffusion-based design also allows integration with multi-omics data (e.g., epigenomic or proteomic layers), offering opportunities to investigate gene regulation under diverse biological or clinical conditions. A promising direction for future work is to extend Planet toward continuous-time modeling, enabling the generation of a series of dynamic GRNs that trace regulatory transitions across disease progression or phenotype changes. Achieving this will likely require integrating additional omics layers, such as epigenomic and proteomic information, to comprehensively reconstruct the temporal regulatory landscape.

## Figures and Tables

**Table 1:** Summary of comparative methods.

Method	Modeling	Data Types	Time Series?
ARACNE	Correlation	Bulk RNA-seq	
CLR	Correlation	Bulk RNA-seq	
DeepSEM	Deep generative models	scRNA-seq	
DigNet	Deep generative models	scRNA-seq	
GENIE3	Decision tree ensembles	Bulk RNA-seq	
GRISLI	Differential equations	scRNA-seq	Yes
PIDC	Information theory	scRNA-seq	
SCENIC	Decision tree ensembles	scRNA-seq	
SINCERITIES	Regression model	scRNA-seq	Yes
SCODE	Differential equations	scRNA-seq	Yes
Tigress	Regression model	Bulk RNA-seq	
MI	Information theory	Baseline	
PCC	Correlation	Baseline	

**Table 2:** Ablation experiment of key modules (average of 10 tests).

	Base	w/o G	w/o C	w/o P	wo G/C	w/o G/P	w/o C/P	w/o GCP
AUROC	0.9136	0.9068	0.9050	0.9074	0.9071	0.8997	0.9084	0.9049
AUPRC	0.8063	0.7862	0.7847	0.7855	0.7955	0.7804	0.7889	0.7850

Notes: w/o G is the isolated GATv2 module; w/o C is the isolated cross-attention mechanism module; w/o P is the isolated feature pooling module.

## Data Availability

The scRNA-seq data of breast cancer can be downloaded from EMBL-EBI ArrayExpress (www.ebi.ac.uk/arrayexpress, accessed on 1 June 2025) under accession number E-MTAB-8107. The mouse aging process dataset can be downloaded from the NCBI Gene Expression Omnibus with accession number GSE247719. The complete source code for Planet is publicly available at https://github.com/wangchuanyuan1/project-Planet (accessed on 12 October 2025). The repository also provides simulated datasets, pre-trained model weights, and step-by-step tutorials to facilitate result reproduction and further research use.

## Supplementary Materials

The following supporting information can be downloaded at: https://www.mdpi.com/article/10.3390/genes16111255/s1. Note S1. Comparative Analysis of Planet and DigNet on the HCC Dataset; Table S1. Detailed information of the HCC test set (hsa05225 KEGG pathway); Table S2. Performance evaluation of Planet and DigNet on hepatocellular carcinoma and normal liver tissue datasets; Figure S1. Comparative network analysis of Planet and DigNet in T cells from the hepatocellular carcinoma dataset. (A) Jaccard similarity and Kolmogorov–Smirnov test results indicating structural divergence between the two generated Gene regulatory networks. (B) Venn diagram depicting the overlap between Planet- and DigNet-derived regulatory networks and the reference gene regulatory network. Reference [46] is cited in the Supplementary Materials.

## Abbreviations

The following abbreviations are used in this manuscript:

GAT	Graph Attention Encoder
GRN	Gene Regulatory Network
scRNA-seq	single-cell RNA sequencing
TF	Transcription Factors
TriHAT	Triple Hybrid-Attention Transformer
