# Supplementary material for: Attention-Guided Probabilistic Diffusion Model for Generating Cell-Type-Specific Gene Regulatory Networks from Gene Expression Profiles
Source: Genes (Basel). 2025 Oct 24;16(11):1255. doi: 10.3390/genes16111255 (PMC12651998; doi:10.3390/genes16111255)
Supplement: Supplementary file 1 [file genes-16-01255-s001.zip › genes-3924925-supplementary.pdf]

Supplemental Materials

Attention-Guided Probabilistic Diffusion Model for Generat-ing Cell-Type-Specific Gene Regulatory Networks from Gene Expression Profiles

Shiyu Xu[1], Na Yu[2], Daoliang Zhang[3] and Chuanyuan Wang [1, 4, *]

[1] Department of Bioinformatics, School of Life Sciences, Xuzhou Medical University, Xuzhou, Jiangsu, China; xushiyu163@163.com (S.X.); cywang@xzhmu.edu.cn (C.W.).

[2] School of Life Sciences, Westlake University, Hangzhou, Zhejiang, China; yuna@westlake.edu.cn

[3] Institute of Science and Technology for Brain-Inspired Intelligence, MOE Key Laboratory of Computational Neuroscience and Brain-Inspired Intelligence, MOE Frontiers Center for Brain Science, Fudan University, Shanghai, China; zhangdaoliang@fudan.edu.cn.

[4] Department of Biomedical Engineering, School of Control Science and Engineering, Shandong University, Jinan, Shandong 250061, China; cywang@xzhmu.edu.cn.

Note S1. Comparative Analysis of Planet and DigNet on the HCC Dataset

To further validate the performance of Planet under diverse biological contexts, we conducted additional benchmarking experiments using a single-cell hepatocellular carcinoma (HCC) and normal liver tissue datasets reported by Sun *et al.* [1] (CNP0000650, CNGBdb). The HCC dataset contains 13,693 cells from 18 individuals, annotated into six cell types (the smallest cluster contains 118 cells). The normal liver tissue dataset encompasses 2,213 cells from 12 individuals, spanning 4 cell types, with the smallest cluster containing 168 cells. All gene expression matrices were filtered to remove cells and genes with more than 95% missing values.

Both Planet and DigNet were trained on the same preprocessed data using the iMetacell algorithm for single-cell integration. Model configurations followed the

original authors' recommendations, with Planet employing additional cross-attention layers to facilitate graph diffusion learning. Training was conducted for 600 epochs, and both models were evaluated on the KEGG pathway subset hsa05225 using NVIDIA RTX A6000 GPUs. **Table S1** lists the details of the testing dataset.

Table S1. Detailed information of the HCC test set (hsa05225 KEGG pathway).

	Number of genes	Number of regulatory relationships in the reference network			
		From RegNetwork	From Pearson correlation	From mutual information	Total (removing duplicates)
T cell	126	518	50	100	628
B cell	125	514	58	112	566
Natural Killer cell	123	508	74	98	554
Myeloid derived cell	129	528	58	126	633
Hepatic stellate cell	142	638	140	134	733
Tumor cell	141	636	134	82	799
T cell (Normal)	126	521	116	104	617
B cell (Normal)	124	509	1247	116	531
Natural Killer cell (Normal)	124	495	124	82	504
Myeloid derived cell (Normal)	124	491	96	84	550

Note: *Normal* represents the cell type in normal liver tissue.

Performance metrics (AUROC, AUPRC, and F1-score) were computed for each cell type and summarized in **Table S2**. Across 30 evaluations, Planet outperformed DigNet in 21 cases, with an average improvement of 1.66%. Despite similar numerical performance, comparative analysis of the inferred T cell regulatory networks revealed substantial topological differences (Jaccard similarity = 0.133, KS test $p = 9.04 \times 10^{-17}$), indicating that the two methods capture distinct aspects of gene regulation (**Figure S1 A-B**).

All preprocessed data, trained models, and generated networks are publicly available via GitHub: https://github.com/wangchuanyuan1/project-Planet and Zenodo: https://doi.org/10.5281/zenodo.17349618 repositories.

Table S2. Performance evaluation of Planet and DigNet on hepatocellular carcinoma and normal liver tissue datasets.

Cell type	Planet AUROC	DigNet AUROC	Planet AUPRC	DigNet AUPRC	Planet F1-score	DigNet F1-score	Δ (%)
T cell	**0.5822**	0.5705	**0.1343**	0.1237	**0.1001**	0.0881	+ 1.1
B cell	**0.5940**	0.5686	**0.1993**	0.0923	**0.1362**	0.0940	+ 5.8
Natural Killer cell	0.5746	**0.5759**	**0.1476**	0.1013	**0.1229**	0.0955	+ 2.4
Myeloid derived cell	**0.5960**	0.5916	0.1375	**0.1456**	**0.1227**	0.0970	+ 0.7
Hepatic stellate cell	**0.6088**	0.5701	**0.2020**	0.0969	0.1015	**0.1263**	+ 4.0
Tumor cell	**0.6052**	0.6030	0.2081	**0.2674**	**0.1351**	0.1159	- 1.3
Tumor cell	**0.6052**	0.6030	0.2081	**0.2674**	**0.1351**	0.1159	- 1.3
T cell (Normal)	**0.6532**	0.6067	**0.2603**	0.1717	**0.1442**	0.098	+ 6.0
B cell (Normal)	0.598	**0.609**	0.1467	**0.1992**	0.1073	**0.1106**	- 2.2
Natural Killer cell (Normal)	**0.6113**	0.6084	0.1814	**0.2083**	**0.1109**	0.0989	- 0.4

Note: Δ(%) is the average performance improvement of Planet compared to DigNet. Δ > 0 indicates that Planet performs better. The best results under the same metric are bolded.

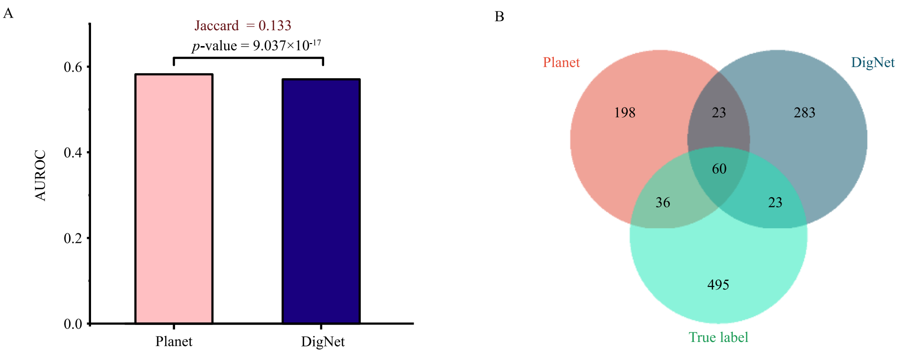

Figure S1. Comparative network analysis of Planet and DigNet in T cells from the hepatocellular carcinoma dataset. (A) Jaccard similarity and Kolmogorov–Smirnov test results indicating structural divergence between the two generated Gene regulatory networks. (B) Venn diagram depicting the overlap between Planet- and DigNet-derived regulatory networks and the reference Gene regulatory network.

Supplementary References

1. Sun, Y.; Wu, L.; Zhong, Y.; Zhou, K.; Hou, Y.; Wang, Z.; Zhang, Z.; Xie, J.; Wang, C.; Chen, D. Single-cell landscape of the ecosystem in early-relapse hepatocellular carcinoma. *Cell* **2021**, *184*, 404-421. e416.
